# Coinfection of disseminated cryptococcosis and BK Virus, a casualty of missed diagnosis during the COVID-19 Pandemic: A case report and review of the literature

**DOI:** 10.18502/cmm.7.2.7802

**Published:** 2021-09

**Authors:** Jéssica Louise Benelli, Rossana Patrícia Basso, Márcia de Lima Rodrigues, Vanice Rodrigues Poester, Lívia Silveira Munhoz, Valerio Rodrigues Aquino, David A. Stevens, Melissa Orzechowski Xavier

**Affiliations:** 1 Laboratory of Mycology, Faculty of Medicine, Federal University of Rio Grande, Rio Grande (FURG), Brazil; 2 Postgraduate Program in Health Science, Federal University of Rio Grande (PPGCS-FURG), Rio Grande, Brazil; 3 Clinical Analysis Laboratory, Dr. Miguel Riet Correa University Hospital, Rio Grande (HU-FURG/EBSERH), Brazil; 4 Specialized Care Service in Infectious Diseases, Dr. Miguel Riet Correa University Hospital, Rio Grande, Brazil; 5 Laboratory Diagnostic Service, Microbiology Unit, Hospital de Clinicas de Porto Alegre, Porto Alegre, Brazil; 6 California Institute for Medical Research, San Jose, and Division of Infectious Diseases and Geographic Medicine, Stanford University School of Medicine, Stanford, California, USA

**Keywords:** BK Virus, Fungal meningitis, HIV-AIDS, Opportunistic diseases, Pandemic

## Abstract

**Background and Purpose:**

The COVID-19 pandemic resulted in an overload of health services and healthcare professionals. The result is a setback in health promotion and prevention, delays in diagnosis, and deaths from other diseases that are currently receiving inadequate attention. This article illustrates the risk of this negligence.

**Case report:**

This study aimed to report a case of coinfection of disseminated cryptococcosis and BK virus in a patient without a previous diagnosis of human immunodeficiency virus infection and COVID-19 negative in the context of the COVID-19 pandemic. Despite receiving antifungal therapy, the patient died.

**Conclusion:**

This fatal case is a warning regarding delay of diagnosis and neglect of other serious illnesses owing to the current pandemic, including fungal diseases and neglected diagnoses.

## Introduction

Coronavirus disease 2019 (COVID-19), declared as a pandemic by the World Health Organization (WHO) in January 2020, has already killed more than 4 million people globally [ 1 ]. The high transmissibility of severe acute respiratory syndrome coronavirus 2 (SARS-CoV-2) and frequent severe COVID-19 illnesses leads to the need to adopt social distancing and isolation measures [ 2 ]. These measures are essential to prevent the collapse of the healthcare system, prolonged isolation, and increased difficulty in accessing health services. However, the massive allocation of resources to COVID-19 has negative impacts on public healthcare strategies [ 3 , 4 ].

Wright et al. in 2020 [ 5 ] referred to the existence of an “invisible epidemic” characterized by diseases that have been neglected as a result of COVID-19, with a proven sudden reduction in screening tests, routine examinations, and preventive medicine [ 5 ]. Included in this epidemic are fungal diseases, such as cryptococcosis, an opportunistic disease responsible for the death of about 15% of patients with the human immunodeficiency virus [ 6 - 9 ].

Its main manifestation is neurocryptococcosis, meningitis caused by fungi of the genus *Cryptococcus*, especially by the species C. neoformans [ 6 ]. This study aimed to report a case of cryptococcosis with late diagnosis, culminating in the progression of the disease and patient death. This is an alert to the medical community about the risk of delaying the diagnostic investigation of other pathogens and diseases during the current COVID- 19 pandemic.

## Case report

A male patient, 37 years old, homosexual, English teacher, was referred to the Specialized Care Service of Infectious Diseases at Dr. Miguel Riet Correa University Hospital, Rio Grande, Brazil on September 8, 2020. He reported fever, enlarged cervical nodes, asthenia, adynamia, and anorexia for 30 days, and moderate-intensity headache that had worsened in the last 10 days. He had several medical visits to external professionals in the preceding 30 days with the same complaints. He had received only symptomatic treatment (dipyrone 500 mg q 6 h) and only investigated for SARS-CoV-2 by reverse transcription-polymerase chain reaction and serological tests. It should be mentioned that the results of both tests had been negative.

**Figure 1:**
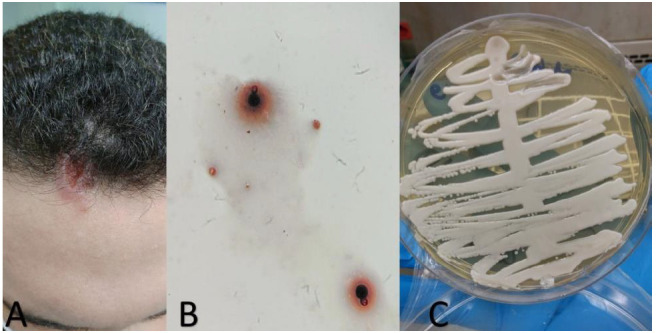
Skin lesion, blastoconidias, and growth of *Cryptococcus* neoformans

On physical examination at Dr. Miguel Riet Correa University Hospital, an ulcerated lesion (2×1 cm) was noted on the face, specifically on the forehead and close to the scalp with irregular borders and fibrinous background that had evolved over 10 days (Figure 1A). Other findings included cervical and submandibular adenopathy with nodes of 1.5-2 cm without oral lesions or other systemic findings. The ensuing workup included blood count; serology for human immunodeficiency virus (HIV), hepatitis B surface antigen, hepatitis C virus, Epstein-Barr virus, cytomegalovirus, and toxoplasmosis; venereal disease research laboratory test; computed tomography of the brain; and a chest radiograph.

Antibodies to HIV were present and other serologic tests were negative. The computed tomography of the brain and the chest radiograph showed no abnormalities. The values of hematocrit, hemoglobin, leukocyte count, segmented neutrophils, and platelets were 36%, 12.7 g/dL, 10,340, 82%, and 367,000, respectively, with preserved renal and liver function. Since the patient had a history of a scratch by a domestic cat with sporotrichosis hyperendemic in the region [ 8 ], a sample of the skin lesion was sent for mycological examination. 

Numerous large and spherical blastoconidia suggestive of *Cryptococcus* species (Figure 1B) were detected in the skin lesion sample, which also resulted in a pure growth of numerous yeast colonies after 48 h of incubation on Sabouraud dextrose agar (Figure 1C). C. neoformans was identified by subculture on niger agar and canavanine-glycine-bromothymol agar and confirmed by MALDI-TOF mass spectrometry with >99% identity. Figure 1. A shows the ulcerated lesion close to the face of the patient, on the forehead, and close to the scalp that had progressed over 10 days. Figure 1. B illustrates the mycological examination with silver (Grocott-Gomori) stain showing spherical blastoconidia in swab material from the skin lesion of the patient, 40X magnification. Figure 1. C shows the yeast-like colony, white to cream, mucoid and shiny, identified as C. neoformans, isolated from the skin lesion of the patient.

On September 10, he had an episode of loss of consciousness and was referred for hospitalization. On admission, the patient was somnolent, with normal pupils, systemic blood pressure of 150/90 mm Hg, heart rate of 60 beats per min, respiratory rate of 19, oxygen saturation of 99%, and no signs of meningeal irritation.

**Figure 2:**
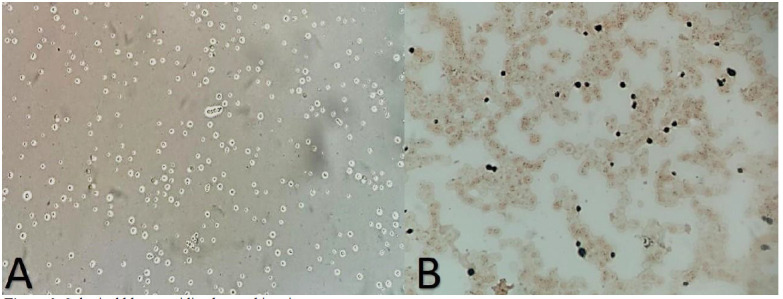
Spherical blastoconidia observed in microscopy

The serum cryptococcal antigen titer was 1:1024 at this time. A lumbar puncture was performed, and an India ink test of the cerebrospinal fluid (CSF) revealed yeasts with morphology characteristic of *Cryptococcus* species (Figure 2A). Moreover, polyomavirus BK virus was detected by real-time polymerase chain reaction. A CSF sample showed an opalescent white aspect with decreased glucose values (17 mg/dL) and increased protein (72 mg/dL) red blood cells (200/mm3), and leukocytes (250/mm3; 95% segmented, 5% lymphocytes). The CSF cryptococcal antigen was also positive (titer>1:1024). According to WHO guidelines and considering the unavailability of flucystosine in Brazil, antifungal therapy started with intravenous amphotericin B (Amb) deoxycholate 50 mg/d and oral fluconazole (Flu) 800 mg/d [ 6 ].

Microscopy examination of blood culture (Figure 2B)and CSF (Figure 2A) showed encapsulated yeasts suggestive of *Cryptococcus* species, confirmed by the growth of C. neoformans on culture, proving the diagnosis of disseminated cryptococcosis. The isolate showed no resistance in vitro to Amb or Flu by microdilution technique (minimum inhibitory concentration of Amb: 1 µg/mL, the minimum inhibitory concentration of Flu: 4 µg/mL). Figure 2. A shows a spherical encapsulated blastoconidia observed in microscopy with India Ink, visualized in the CSF sample of the patient with 40X magnification. Figure 2. B illustrates a large, spherical blastoconidium in direct examination of blood culture (Grocott-Gomori stain) with 40X magnification.

Over the next 24 h, his course evolved with disorientation, left deviation of the mouth and palpebral ptosis with heart rate, respiratory rate, and systemic blood pressure of 117, 22, and 90/ 60 mm Hg, respectively. Finally, he was transferred to the Intensive Care Unit. 

On September 12, the patient was vomiting and consequently aspirated gastric contents. He had anisocoria with left mydriasis and sensorium lowering that progressed to a comatose state rated 3 on the Glasgow scale. A new CT scan of the brain was requested, revealing generalized edema, diffuse ablation of cerebral sulci and fissures, and slightly hyperdense dural sinuses, possibly related to cerebral venous thrombosis. Nodular thickening of the skin and subcutaneous tissue in the right frontal region was seen, corresponding to the area of the skin lesion. The patient required orotracheal intubation and treatment was started with cefepime 1 g/8h IV and clindamycin 600 mg/8h IV for aspiration pneumonia. It should be mentioned that aspiration material was not collected for analysis.

**Figure 3:**
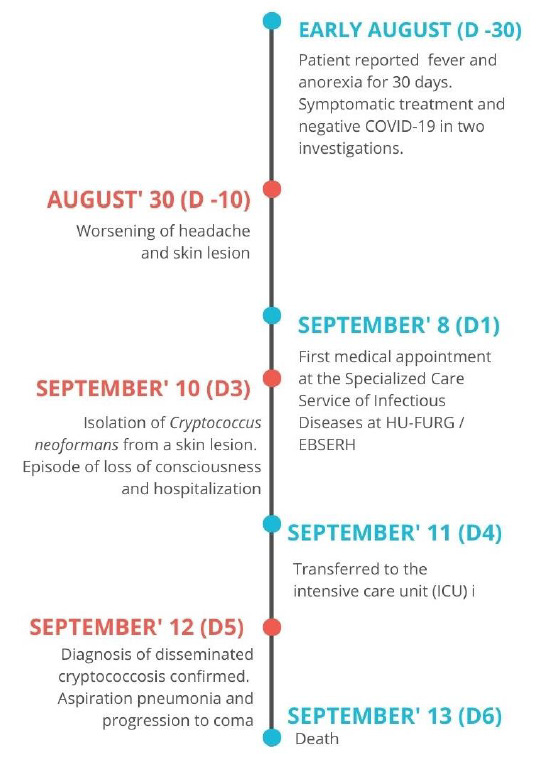
Timeline of the progression of the case.

The patient died within 48 h after admission to the Intensive Care Unit, on September 13. The HIV viral load tests and CD4+ count were requested but their results were inconclusive. No other test was performed due to his rapid deterioration (6 days between hospitalization and death) (Figure 3). Figure 3 shows a timeline of case progression, from the onset of symptoms to death.

## Discussion

This study presents a case of disseminated cryptococcosis in a patient with HIV infection, which had evolved for more than 30 days before the diagnosis of this viral-fungal co-infection. In the context of the COVID-19 pandemic, his full diagnostic investigation was neglected, resulting in severe disease progressing to death. The patient lacked a severe headache (headache is common in neurocryptococcosis) and did not have a prior diagnosis of HIV infection. 

A skin lesion with a high load of fungal propagules was the first important evidence of a disseminated presentation of cryptococcosis. These cutaneous manifestations occur in only ~5% of cases and are generally associated with low T CD4+ cell counts [ 9 , 10 ]. With the relatively insidious onset, he was only diagnosed at the moment of its sudden progression to a severe and irreversible condition. The patient died due to the advanced stage of the disease, even with supportive therapy and antifungal treatment, reaffirming the impact of the late diagnosis of cryptococcosis on the unfavorable outcome in HIV/ acquired immunodeficiency syndrome (AIDS) patients [ 6 , 7 ]. However, prophylaxis with Flu is not recommended due to its inefficiency in preventing cryptococcosis and the risk of inducing resistance, highlighting that an early diagnosis is the most efficient “prophylactic measure” for cryptococcosis [ 6 , 8 ].

Despite the absence of data on viral load and CD4+ counts, the evolution of the condition suggests a high degree of immunosuppression, a hypothesis supported by the skin lesion with high fungal burden, and by the detection of BK virus DNA in the cerebrospinal fluid of the patient [ 13 , 14 ].

The BK virus (BKV) is a pathogen of the family of polyomaviruses, and its infection is quite common with a prevalence of up to 90% antibodies in adults. It is also found in the whole blood sample of about 30% of HIV patients [ 13 , 14 ]. The BKV usually causes asymptomatic infection in children; however, the virus is latent in the body, being reactivated in immunodeficient individuals [ 14 ], especially when there is depletion of cellular immunity when the presence of viral DNA is associated with depletion of CD4+ cells [ 15 ].

Infection with BKV in an HIV patient can rarely lead to progressive encephalopathy [ 14 ]. In this case, it may have contributed to the worsening of the symptoms and signs found on his brain CT. The BKV was isolated from neurological tissue. It is rarely reported in the literature [ 16 , 17 ]; however, there are no prior reports of co-infection of *Cryptococcus* species and BKV affecting the central nervous system. We found only three reports of co-infection of C. neoformans and BKV, all in transplanted patients with BKV causing kidney injury (Table 1) [ 18 - 20 ].

**Table 1:** Brief literature review of Cryptococcus sp. and BK-Virus co-infection case reports

References/ Data	[18]	[19]	[20]	Case report
Sex	Man	Man	Woman	Man
Age	40	31	30	37
Location	India	United States of America	South Africa	Brazil
Base disease	Renal transplantation	Primary B-lineage acute lymphoblastic leukemia with HSCT	Transplanted kidney	HIV/AIDS
Others Comorbidities	Hepatitis B	*Pseudomonas aeruginosa* pneumonia and bronchial culture with *Mycobacterium avium* at 100 first days of HSCT	Recurrent urinary tract infection and renal disease, a complication of previous malarial illness, and CMV infection	HIV/AIDS without previous diagnosis, viral load and CD4+ unknown.
Imunossupression	Yes	Yes	Yes	Yes
Type of Cryptococcosis	Pulmonary	Disseminated with renal involvement	Cryptococcoma in transplanted kidney	Disseminated with skin lesion
Cryptococcosis diagnosis	Histopathological	Antigen detection and positive cultures in the blood, pleural fluid and cerebrospinal fluid	Radiological and histopathological appearances	Antigen detection and positive cultures from the skin lesion, blood, and cerebrospinal fluid
BK- Virus infection	Nephropathy	Nephropathy	Nephropathy	Central nervous system
BKV diagnosis	Histopathological and plasma detection by molecular testing	Plasma and urine detection by molecular testing	Urine detection by molecular testing	Cerebrospinal fluid detection by molecular testing
Treatment for Cryptococcosis	First: Amphotericin (6 weeks) and Fluconazole prophylaxis Later: Amphotericin and Flucytosine.	Liposomal amphotericin B 5 mg/kg/day and flucytosine 25 mg/kg/day for 3 weeks Later: Twice daily oral voriconazole 200 mg and continued on 4 mg/kg of IV L-AMB thrice weekly for an additional 6 weeks	Fluconazole 400 mg daily with the intention of continuing to 12 months. Later: Treatment for the cryptococcosiswas escalated by adding 200 mg of fluconazole after each dialysis session.	Amphotericin B deoxycholate 50 mg/d and fluconazole 800 mg/d
Outcome	Discharged	Comfort care measures	Death	Death

Lovati et al. in 2020 emphasized that neurological and systemic symptoms cannot be ignored and require further investigation even within the context of COVID-19. They reported a case of a late diagnosis of herpes virus encephalitis in an elderly person due to the delay in the doctor's search “for fear of COVID-19”, and by an exclusive investigation of the SARS-CoV-2 virus infection in the patient without considering other options [ 21 ].

The reduction in outpatient visits due to the fear of exposure to environments contaminated by SARS-CoV-2 and the diversion of medical resources to deal with the pandemic make the correct and timely diagnoses of other diseases difficult. This is true in the case of diseases with fatal consequences, such as AIDS and cryptococcosis as in our patient. This increases the number of (indirect) victims of COVID-19, due to errors or diagnostic failures [ 21 ].

Tili et al. in 2020 affirmed that the pandemic culminates due to some medical negligence and inequalities that delay or even paralyze the health services and care for other acute and chronic conditions [ 22 ]. The total effects on public health are immeasurable, especially in countries with a large part of the population living in vulnerable situations, in poverty, or without access to health services [ 4 ]. Gomolim et al. in their study performed in 2020 also warned us regarding the danger of neglecting diseases that can be prevented or benefit from an early diagnosis, such as melanoma and other types of cancer [ 3 ].

## Conclusion

Cryptococcosis is on the list of neglected diseases of WHO, even though it is among the five most lethal diseases [ 6 ]. Late diagnosis is still the leading cause of death from cryptococcosis in developing countries [ 6 - 8 ]; however, in our region, diagnostic tests are available, highlighting the necessity of awareness of the risks associated with the negligence of this kind of diagnosis. 

The reported case is another victim of this severe opportunistic mycosis, probably due to its late diagnosis, with a limited prior investigation restricted to COVID-19. Even with treatment, the lethality rate in cryptococcosis is within the range of 20 and 50% [ 23 ]. Therefore, the danger in neglecting systemic diseases with a progressive evolution may result in worsening the prognosis and possible lethality.

## Acknowledgments

The authors are grateful to the Health Science Post-graduation program, College of Medicine from the Federal University of Rio Grande, Rio Grande, Brazil, Coordination for the Improvement of Higher Education Personnel, Brazil, and the Capes PrInT–FURG Program (Institutional project for the internationalization of the Federal University of Rio Grande).

## Funding

This project does not have specific funding for its conduction.

## Authors’ contribution

J. L. B., V. R. P., L. S. M., M. O. X., and V. R. A. performed the sampling and conducted the diagnosis and identification tests. J. L. B. and R. P. B. wrote the first draft of the manuscript. J. L. B., V. R. P., L. S. M., M. L. R., and R. P. B. contributed to the conduction of the study and data preparation. R. P. B. and M. L. R. contributed to care and monitoring of the patient and researching the clinical information. D. A. S. and M. O. X. managed the project, analyzed the data, and finalized the manuscript. All authors had full access to all the data in the study and take responsibility for the integrity of the data and the accuracy of the data analysis.

## Conflicts of interest 

The authors declare that there was no conflict of interest in this study.

## Financial disclosure

No financial interests have been declared related to the material of this manuscript. 

## Financial disclosure

This project was approved by the Health Research Ethics Committee of the Universidade Federal do Rio Grande (number: 234/2018).
